# Evaluation of Extracurricular Medical Education in Cardiothoracic Surgery and Cardiology; Students’ Opinion On Current Medical Training

**DOI:** 10.1007/s10916-023-01988-3

**Published:** 2023-09-01

**Authors:** Sulayman el Mathari, Aïmane Arrouby, Noor Boulidam, Jolanda Kluin

**Affiliations:** Department of Cardiothoracic Surgery, Amsterdam University Medical Center, Amsterdam, The Netherlands

**Keywords:** Extracurricular education, Cardiothoracic surgery, Cardiology, Medical education

## Abstract

Medical students in the Netherlands have a relatively low exposure to cardiothoracic surgery and cardiology. This results in discrepancies between starting levels of medical residents and expected standards in clinical practice. Another side effect is the international decline in interest in a career in these cardiovascular fields. To compensate for this, extracurricular education programs focused on cardiothoracic surgery and cardiology have been set up. This study aims to evaluate the impact and benefits of an extracurricular education program on students' knowledge of cardiothoracic surgery and cardiology and to explore possible factors involved in (academic) career decision making. 66 participants were enrolled in the extracurricular education program VECTOR and were included in a cross-sectional cohort study. All participants scored their familiarity with different aspects of cardiothoracic surgery and cardiology prior to and after the course. Data was analyzed using Linear regression and Wilcoxon Signed Rank testing. Participants demonstrated a significant trend towards improved familiarity with cardiothoracic surgery and cardiology after the program (*p* = < .001). Significant increased knowledge scores were reported for all specified subdomains after the course with an increase in academic interest in cardiology (*p* = < .001) and cardiothoracic surgery (*p* = < .001). Students revealed that clinical responsibilities and a healthy work-life balance were considered most important when deciding upon their future career. This study highlights the benefit of an extracurricular education program in cardiothoracic surgery and cardiology for undergraduates and could possibly aid students in preparing for clinical practice.

**Supplementary Information:**

The online version contains supplementary material available at 10.1007/s10916-023-01988-3.

## Introduction

Starting medical residents are amongst the first healthcare professionals to provide initial hospital care in the challenging medical specialties of cardiothoracic surgery and cardiology. Therefore, adequate basic knowledge of this population is essential to guarantee quality of care.

In the Netherlands, medical training starts with 6 years of study in medicine. The first 3 years are focused on foundational knowledge of all medical specialties. The last 3 years consist of clinical rotations and research. In the whole curriculum of medical school, only 5% of all training is spend on cardiovascular medicine [1]. Furthermore, content during this exposure remains superficial to fit in the overarching education program. Basic understandings of cardiology are provided over 9 weeks of teaching and cardiothoracic surgery is not included in the standard medical program.

This affects both the interest of students in cardiovascular medicine and the starting level of beginning medical residents. For example, student interest in pursuing a career in cardiothoracic surgery has been declining internationally for several years now [2, 3], and starting medical residents are beholden to practice a significant amount of self-study to meet the standards in the field of cardiovascular medicine [4, 5].

To fill the gap between knowledge provided in medical school and the obvious higher standards in clinical practice, several initiatives have been taken by students and young physicians facilitating extracurricular education in cardiothoracic surgery and cardiology. One of these initiatives is VECTOR [6]. VECTOR is a Dutch student association for extracurricular cardiovascular education supervised by cardiothoracic surgeons and cardiologists. In this program, students participate in a range of interactive lectures and hands-on practicals, during which they are exposed in-depth to both cardiothoracic surgery and cardiology.

Earlier studies have shown the benefits of extracurricular courses in plastic surgery, medical anatomy programs and nursing education [7–9]. However, there are no studies on the effects of undergraduate extracurricular education in cardiothoracic surgery and cardiology. Therefore, we performed a study to describe the effect and added value of extracurricular education in cardiothoracic surgery and cardiology among undergraduates.

## Methods

This is a cross-sectional study. 66 participants were included, that attended the VECTOR extracurricular course during the academic year 2021–2022. All participants were asked to fill in a custom questionnaire at the end of the program, consisting of 10 statements. This non-validated questionnaire can be found in Supplemental A. Participants were instructed to express their agreement with the statements on a 6 point Likert scale [10]. Contents of the used statements were constructed to reflect different aspects of the regular undergraduate curriculum in relation to the extracurricular program. Student’s knowledge, practical skills and interest in specific topics were assessed. In addition, demographic data such as age, biological sex and year of study were collected.

Statistical analyses were performed using IBM SPSS Statistics 28.0 software for Windows. Categorical demographic data is reported as frequencies and percentages, whilst non-normally distributed continuous variables are displayed as a median with corresponding interquartile range. Using the Likert-scale, students indicated their familiarity with the topics in the course. Data from the questionnaire was considered ordinal with each category representing a different value ranging from 1 to 6 (1 = lowest, 6 = highest). The higher the score, the greater the student’s insight in this topic. The overall effect of the extracurricular program was evaluated using a Linear Regression analysis. In addition, sub-analyses were performed using Wilcoxon Signed Rank testing. For all analyses, results were considered statistically significant if *p* < 0.05.

Written informed consent was collected from all patients. All data remained anonymous and participation was completely voluntary.

## Results

Data from 66 participants taking part in the VECTOR program was collected. Baseline characteristics can be found in Table 1. Of 66 participants, 56,1% was female. Median age of the participants was 21 years. Regarding study progress at the start of the program, 19.7% was a first year student, 22.7% second year student, 34.8% third year student, 18.2% fourth year student and 4.5% fifth year student.

**Table 1:** Baseline characteristics of VECTOR participants

**Demographic** **Characteristics**	**Frequency** **(N = 66)**
**Age**	
Average (*IQR*)	21.00 (*3*)
**Sex**	
Male (*%*)	29 (*43.9*)
Female (*%*)	37 (*56.1*)
**Level of study**	
Year 1 (%)	13 (19.7)
Year 2 (*%*)	15 (*22.7*)
Year 3 (*%*)	23 (*34.8*)
Year 4 (*%*)	12 (*18.2*)
Year 5 (*%*)	3 (*4.5*)

### Overall impact of the course

For all participants, cumulative scores for all statements were calculated prior to and after the course. This included scores related to student’s knowledge about cardiothoracic anatomy, common cardiovascular diseases, ECG interpretation, clinical duties of cardiovascular specialists and relevant (scientific) developments within the field of cardiothoracic surgery and cardiology. Participants demonstrated significantly increased scores after participating in the program (*B* = 6.89 [5.80 – 7.99]; *p* =  < 0.001; R^2 = 0.55).

Linear regression analysis with adjustments for sex revealed no significant difference in overall scoring between males and females (*p* = 0.348).

To investigate if study year was a significant effect modifier in this association, participants were dichotomized into two groups: Bachelor students (year 1, 2 and 3) and Master students (year 4 and 5). Based on the p-value of the corresponding interaction term, discrepancies in study year did not result in significant differences in Likert-scale values after the course (*p* = 0.29). After adjusting for sex and study-year, participants still demonstrated a significant trend towards improved knowledge standards after the program (*B* = 6.89 [5.82 – 7.97]; *p* =  < 0.001; R^2 = 0.57), implying that the program contributed to a significant increase in cardiovascular knowledge regardless of study year at baseline.

### Impact of the programme per course-domain

**Fig. 1:**
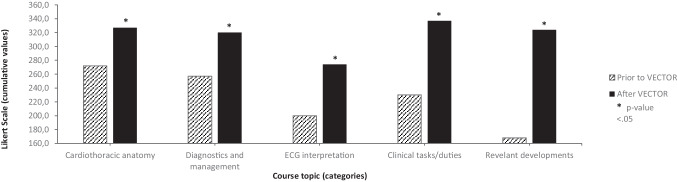
Cumulative scores for VECTOR course topics, prior to and after the VECTOR program. For all course topics, a significantly higher value was given with regards to familiarity relating to the topic after following the course when compared to prior to the course

Cumulative scores for all aspects of the program are visualized in Fig. 1. Cardiothoracic anatomy (*Mdn* = 4), common cardiovascular disease (*Mdn* = 4) and clinical duties of cardiovascular specialists (*Mdn* = 4) appeared to be more familiar topics prior to the program compared to interpretation of ECG’s (*Mdn* = 3). and relevant developments within cardiovascular medicine (*Mdn* = 2).

Differences in student’s knowledge prior to and after the program are presented in Table 2. Wilcoxon Signed Rank testing revealed that students attributed significantly higher scores after the program for all subdomains of the course. Students improved all of their scores with significant effect sizes (*r* > 0.50). Largest improvement was shown in awareness of relevant developments within the field (*Mdn* = 5; *p* =  < 0.001; *r* = 0.62) followed by knowledge on common cardiovascular diseases and their appropriate diagnostics and management (*Mdn* = 5;* p* =  < 0.001; *r* = 0.58). Students also significantly improved their ability to interpret ECG’s (*Mdn* = 4;* p* =  < 0.001; *r* = 0.56) and showed similar advancements in knowledge about cardiothoracic anatomy (*Mdn* = 5;* p* = 0.001; *r* = 0.50) and clinical duties of medical specialists (*Mdn* = 5;* p* = 0.001; *r* = 0.56).

**Table 2:** Wilcoxon Signed Rank Test for student’s cumulative scores for VECTOR course topics, prior to and after the VECTOR program

**Domain**	***p-value***	***z-value***	***Effect size (r)***
Cardiothoracic anatomy	< 0.001	-5.72	0.50
Common cardiovascular diseases including diagnostics and management	< 0.001	-6.72	0.58
ECG interpretation	< 0.001	-5.98	0.52
Clinical tasks / duties of the cardiologist and thoracic surgeon	< 0.001	-6.48	0.56
Relevant developments within the field of cardiology and cardiothoracic surgery	< 0.001	-7.07	0.62

Results were considered statistically significant when p-value < 0.05

### Academic interest

**Fig. 2:**
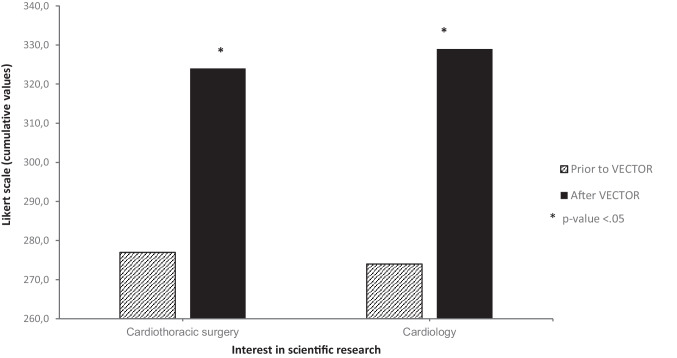
Student interest in scientific research in both cardiothoracic surgery and cardiology significantly increased when comparing prior to and after the VECTOR program

Differences between interest in scientific research prior to and after the program are visualized in Fig. 2. Cumulative scores derived from the Likert-scale are presented separately for cardiothoracic surgery and cardiology. Participants showed a comparable interest for cardiothoracic surgery and cardiology (total score = 324; *Mdn* = 5 and total score = 329; *Mdn* = 5).

Wilcoxon Signed Rank testing demonstrated that Likert-scale scores related to pursuing an academic career had significantly increased for both cardiothoracic surgery (effect size r = 0.45) and cardiology (effect size r = 0.53) after the program (Table 3).

**Table 3:** Wilcoxon Signed Rank Test for interest in scientific research in cardiothoracic surgery and cardiology prior to and after the VECTOR program

**Interest in scientific research**	***p-value***	***z-value***	***Effect size (r)***
Cardiothoracic surgery	< 0.001	-5.22	0.45
Cardiology	< 0.001	-6.10	0.53

Results were considered statistically significant when p-value < 0.05

### Factors involved in career decision-making

**Fig. 3:**
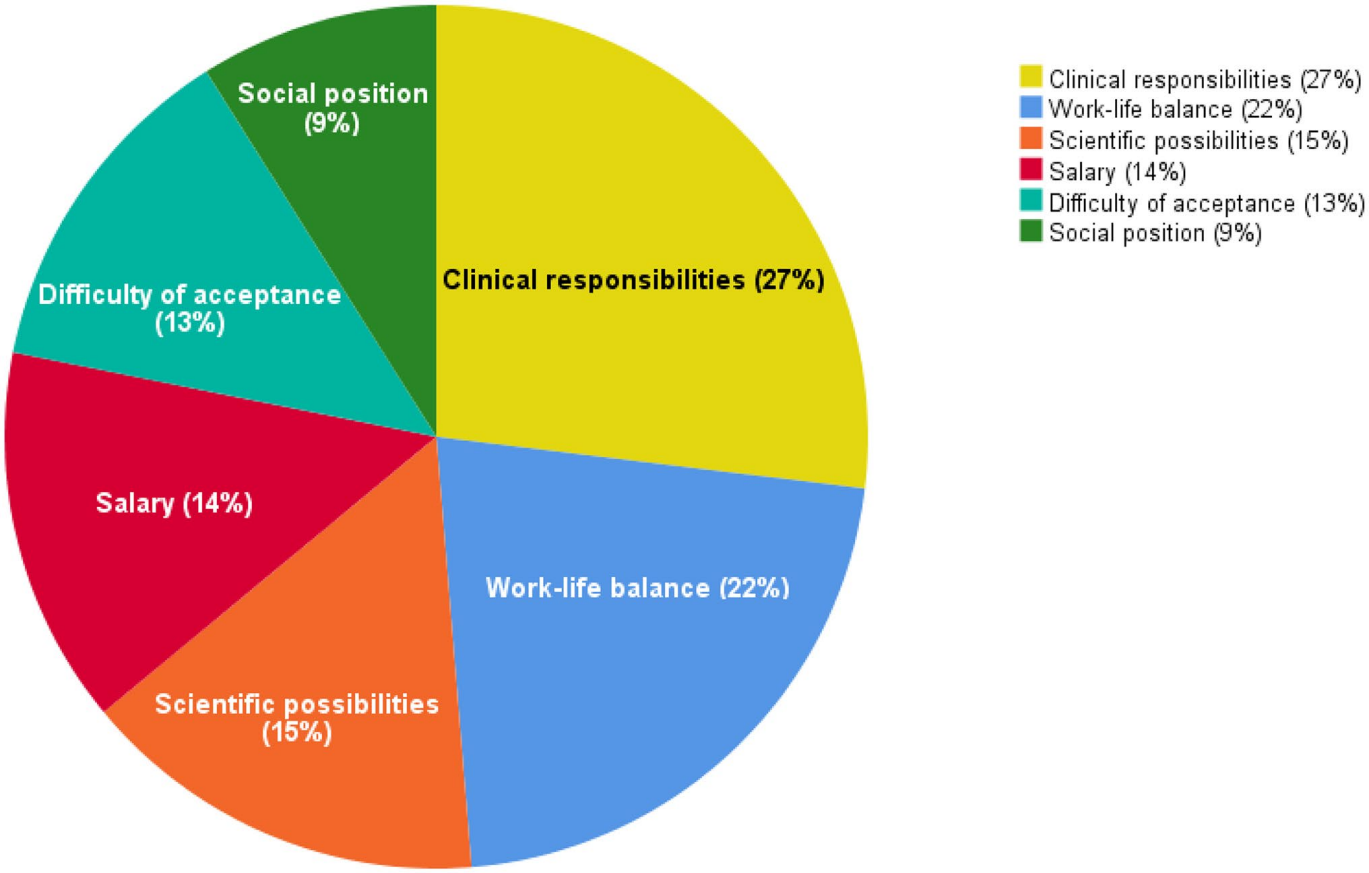
Importance of factors involved in career decision-making according to medical students participating in VECTOR program. Clinical responsibilities (27%) and work-life balance (22%) were scored highest, followed by scientific possibilities (15%) and salary (14%). Difficulty of being accepted into a residency program (13%) and social status (9%) were deemed least important

When asked which factors are important in deciding upon their future career, students revealed that clinical responsibilities and a healthy work-life balance were considered the most important elements. These two factors combined account for 49% of the decision (clinical duties = 27%; work-life balance = 22%). Whether the profession would offer an appropriate salary (14%) or whether it was possible to pursue an academic career (15%) seemed less important. Social status (9%) and difficulty of being accepted into a residency program (13%) were considered least important in decision-making. Results are displayed in Fig. 3.

## Discussion

This study shows the benefits of an extracurricular education program in cardiothoracic surgery and cardiology for undergraduates. A significant increase was seen in all specified course topics after following the program, ranging from anatomical knowledge to etiology, diagnostic methods and (scientific) developments in the respective fields. Linear regression analysis showed that the increase in score was universal when stratified by study phase (bachelor, master), highlighting the benefit of this program regardless of study year at baseline. There were no significant differences in scores between men and women.

Students reported a significant increased interest in scientific research in cardiothoracic surgery and cardiology after following the program. This showcases a potential advantage for departments of cardiothoracic surgery and cardiology to attract talented and ambitious undergraduates for clinical research.

With regards to declining interest in cardiothoracic surgery in the past years, medical departments might be interested to learn what factors influence students to choose a career path. Our findings show a strong preference towards clinical duties and a healthy work-life balance. This suggests that departments might benefit from diverting their recruiting efforts by providing students with a clearer understanding of what their specialty entails. Also, students may be more interested in pursuing a career in cardiothoracic surgery or cardiology when a decent work-life balance can be attained. Salary and academic opportunities also seem to be of importance in career-decision, but to a lesser extent than the aforementioned factors.

The most important limitation in this study is the limited number of participants. For this reason, regression analysis was performed using dichotomized variables for study year to decrease the margin of error and increase the study power. Another limitation involves the self-reported outcome measures by participants which might have influenced results. Participants completed the questionnaire after following the course. As this questionnaire contained elements of the participants’ own perceived knowledge prior to the course, it is likely that this bias affected scores at baseline. However, the cross-sectional design of our study might have improved score accuracy, as students gained better insight of what they did not know prior to the course. This cognitive bias phenomenon is known as the Dunning-Kruger effect [11]. This might have facilitated a more appropriate estimation of students’ knowledge compared to a before-and-after design.

Moreover, this study employed a non-validated questionnaire to gather data about participants' engagement in the extracurricular program. The goal was to assess the program's effects on students' knowledge, practical skills, and interest in cardiology and cardiothoracic surgery. Although the questionnaire was customized to address the relevant research questions, this approach could have introduced limitations with regards to result interpretation. It's worth noting that validated questionnaires tailored for medical education are currently lacking. To improve the robustness of findings, future investigations could bolster methodological precision by formulating and validating a questionnaire aligned with specific study parameters in this context.In the future, a longitudinal study is needed with a larger number of participants that explores students’ interest in cardiothoracic surgery and cardiology over the years and follows their career choices. This will display long term results and confirm potential benefits of an extracurricular educational program in cardiothoracic surgery and cardiology.

In conclusion, our findings suggest a beneficial effect of an extracurricular education program in cardiothoracic surgery and cardiology for undergraduates. We recommend departments of cardiothoracic surgery and cardiology and university faculties to implement a comparable program in their standard curriculum to motivate talented and ambitious students towards the field of cardiovascular medicine and improve the starting level of clinical care in cardiothoracic surgery and cardiology among beginning medical residents.

### Supplementary Information

Below is the link to the electronic supplementary material.

Supplementary file1 (PDF 113 KB)

## Data Availability

The data that support the findings of this study are available from the corresponding author (S.M), upon reasonable request.
