# Supplementary material for: Evaluation of Extracurricular Medical Education in Cardiothoracic Surgery and Cardiology; Students’ Opinion On Current Medical Training
Source: J Med Syst. 2023 Sep 1;47(1):96. doi: 10.1007/s10916-023-01988-3 (PMC10473978; doi:10.1007/s10916-023-01988-3)
Supplement: Supplementary file 1 — Supplementary file1 (PDF 113 KB) [file 10916_2023_1988_MOESM1_ESM.pdf]

Supplemental A

VECTOR education program questionnaire

**Questions**	**Score (1 - 6)**
1. The teaching I have received in medical school in cardiovascular medicine has been adequate	1 2 3 4 5 6
2. **Prior** to this program, my knowledge about the following subjects were adequate	
- *Anatomy of the thorax*	1 2 3 4 5 6
- *Common cardiovascular diseases, diagnostics and management*	1 2 3 4 5 6
- *ECG interpretation*	1 2 3 4 5 6
- *Clinical duties as an attending*	1 2 3 4 5 6
- *New development in the fields*	1 2 3 4 5 6
3. **After** following this program, my knowledge about the following subjects were adequate	
● *Anatomy of the thorax*	1 2 3 4 5 6
● *Common cardiovascular diseases, diagnostics and management*	1 2 3 4 5 6
● *ECG interpretation*	1 2 3 4 5 6
● *Clinical duties as an attending*	1 2 3 4 5 6
● *New development in the fields*	1 2 3 4 5 6
4. The lecture-based teaching I have received in cardiovascular medicine at VECTOR has been adequate	1 2 3 4 5 6
5. The clinical teaching I have received in cardiovascular medicine at VECTOR has been adequate	1 2 3 4 5 6

6. The amount of attention that has been spent on *cardiothoracic surgery* in this program was satisfactory	1 2 3 4 5 6
7. The amount of attention that has been spent on *cardiology* in this program was satisfactory	1 2 3 4 5 6
8. It would be useful if this depth of cardiovascular knowledge would be the norm in medical school	1 2 3 4 5 6
9. **Prior** to this program, I score my interest in pursuing a clinical/ academic career in cardiothoracic surgery as:	1 2 3 4 5 6
10. **After** following this program, I score my interest in pursuing a clinical/ academic career in *cardiothoracic surgery* as:	1 2 3 4 5 6
11. **Prior** to this program, I score my interest in pursuing a clinical/ academic career in *cardiology* as:	1 2 3 4 5 6
12. **After** following this program, I score my interest in pursuing a clinical/ academic career in *cardiology* as:	1 2 3 4 5 6
13. Rank the following factors in order from 1 (most important) to 6 (least important) with respect to their importance in choosing your career.	● Work-life balance<br>● Salary<br>● Social status<br>● Academic possibilities<br>● Clinical duties<br>● The difficulty of getting into a residency program
